# ‘Can They Take the Heat?’—The Egyptian Climate and Its Effects on Livestock

**DOI:** 10.3390/ani12151937

**Published:** 2022-07-29

**Authors:** Amira A. Goma, Clive J. C. Phillips

**Affiliations:** 1 Faculty of Veterinary Medicine, Alexandria University, Alexandria 21944, Egypt; 2 Institute of Veterinary Medicine and Animal Sciences, Estonian University of Life Sciences, Kreutzwaldi 1, 51006 Tartu, Estonia; clive.phillips@curtin.edu.au; 3 Curtin University Sustainability Policy Institute, Kent St., Bentley, WA 6102, Australia

**Keywords:** climate change, Egypt, livestock, heat stress, behaviour, production

## Abstract

**Simple Summary:**

Climate change is receiving more consideration worldwide, and the impact on animal production is particularly relevant due to increasing demand and limitations to production. The location of Egypt means that it has special climatic considerations. In summer, temperatures regularly exceed 40 °C, particularly in lower Egypt, and extreme climatic conditions are resulting from the warming of the planet. Regional temperature and humidity data were used to determine that Egyptian livestock experience heat stress for extended periods in summer. When predicted temperature/humidity increases as a result of climate change are added, the proportion of time that they are exposed to heat stress becomes more severe. It was concluded that the effects of heat stress are becoming critical for livestock production systems, especially during summer. Livestock responds to these changes by using different mechanisms to survive, but production efficiency is severely compromised by heat stress. Injury and death may even ensue if mitigation measures are not taken quickly. Finally, alternative solutions were suggested to help to achieve food security in Egypt.

**Abstract:**

Egypt already has conditions in summer that cause heat stress for livestock, and predicted climate changes suggest that these will be exacerbated over the course of this century. As well, extreme climatic events make the mitigation of climate change difficult but important to understand. Apart from neonates, farm animals have upper critical temperatures in the region of 25–30 °C, whereas temperatures in summer regularly exceed 40 °C for prolonged periods. The temperature and humidity data were collected half hourly to calculate Temperature Humidity Indices and demonstrate that Egyptian livestock at two centers of livestock production in the country would experience heat stress in summer for extended periods of each day. The impact of rising temperatures on livestock in Egypt was reviewed, where extensive resources to mitigate the impact are not often available. It was found that, although there are some prospects to mitigate some heat stress, by using agroforestry systems of production for example, these are unlikely to have a major impact, and reduced food security may ensue over the course of this century.

## 1. Introduction

Climatic condition change means the change in different elements of weather, such as temperature, relative humidity, wind, and rainfall, over a long period of time [1]. The focus is mainly on temperature [2]. In the last century, there was a global increase in the average temperature by 0.7 °C [3]. However, there has been an increase in global temperature change/century to 1.8–4 °C in the 21st century [4]. The responsibility for the temperature increase lies mostly with greenhouse gases (GHG), especially carbon dioxide (CO_2_) and methane (CH_4_), according to the Intergovernmental Panel on Climate Change [5]. Livestock is considered both a contributor and a victim [3].

Egypt can be divided into four geographical climatic regions—Delta, Eastern desert, Western desert, and Sinai [6]. The climate varies from the Mediterranean in the coastal area to hot in the Upper region. The winter season is from December to February and the summer from June to August [7].

The climate change impact on Egypt stems from three issues, sea level rise, water crisis, and increased temperature, necessitating a change in the agricultural strategy [8,9,10,11]. In order to maintain food security and a viable agricultural sector, Egypt must formulate an urgent plan to avoid troubles that might be expected to result from these changes [12].

Central to developing Egypt’s agricultural strategy to deal with climate change is addressing the pressure on livestock production due to the temperature increase and more extreme events [5]. These changes were found to negatively impact livestock performance, even though livestock production is increasing worldwide in response to a growing demand [13]. Reduced performance derives from reduced feed intake, reduced opportunities for grazing, reduced feed quality, and exposure of livestock to heat stress and diseases [14]. The precise impact of heat stress on animal production [15], depends on animal species, husbandry type, and animal body condition [16].

Opportunities to mitigate the impact of gaseous emissions from livestock include technical and managemental changes, as well as the integration of livestock into broader environmental protection [14]. It also requires ecological, social, and economic adjustment to reduce the negative impacts or enhance the positive effects of climate change [17]. Natural adaptation by livestock occurs due to animals’ coping mechanisms, such as coat adaptations. However, because of the speed of anthropogenic climate change, humans have to help animals cope with climate change in order to maintain performance [18]. Human strategies for adaptation include breeding modifications, in addition to changes in policies, e.g., drought mitigation [17]. Therefore, there are three important adaptation components, animal responses, human management changes, and the development of resources [18]. Thus, mitigation of the livestock environment, the adaptation of the feeding systems, and reduction of the number of animals to maintain are key strategies to cope with the thermal stresses to which livestock are exposed.

## 2. Thermal Zones and Limits

Thermoregulation is the ability of the animal to maintain its body temperature by balancing heat gain and heat loss. Above the upper and below the lower critical temperatures (Table 1), animals must increase metabolic heat production to control their core body temperature [19,20,21,22,23,24]. Within these two limits, a thermoneutral zone is considered to be the physiological range within which biological function has limited variability and heat is lost from the skin primarily by non-evaporative mechanisms—conduction, convection, and radiation [25]. There are internal and external factors that affect the thermoneutral zone, including the genotype, species, and physiological status as internal factors, and relative humidity, ambient air velocity, and solar radiation degree as external factors [26].

Above the upper critical temperature, heat is lost by evaporative cooling, through the skin and respiratory tract, and the animal is said to be heat stressed. In humid conditions, an animal’s ability to lose heat by evaporative methods is reduced. As well as the thermoneutral zone, some biometeorologists define a thermal comfort zone with narrower limits, in which animal behaviour is not changed to combat low or high temperatures, e.g., by huddling, orientation towards the sun, etc. Others have defined a ‘prescriptive zone’ where animals may evoke evaporative cooling just during the hotter parts of the day, depending on whether they can lose heat at other times by non-evaporative mechanisms [27]. In some forms of livestock transport, e.g., long-distance shipping, there is limited opportunity for this due to fairly consistent circadian temperatures on the ship. As well, a ‘tolerance zone’ is defined, above and below which core body temperature (CBT) will increase and decrease, respectively, and at CBT extremes, a ‘survival zone’, above and below which an animal will die.

The upper limit of the thermoneutral zone is about 26 °C dry bulb temperature for most farm animals, except neonates, which will be able to tolerate higher temperatures. This temperature will be reduced when relative humidity is >50% [28]. The optimal climatic condition for livestock has been defined as an ambient temperature of 13 to 20 °C, wind velocity of 5 to 18 km/hr, relative humidity of 55 to 65%, and a moderate level of light [29]. The severity of heat stress depends mainly on dry bulb temperature and humidity, which can be combined into a THI temperature–humidity index [30]. There are different methods of calculation of the THI on which categorization of stress level depends. Firstly, the temperature measured in °F is used in this formula: THI = db °F − {(0.55 − 0.55RH) (db °F − 58), db: dry bulb temperature in °F; RH: relative humidity (RH%/100). The American University [31] used this formula to categorize THI values for cattle, buffaloes, sheep, and goats as follows: <72 = absence of heat stress, 72 to <74 = moderate heat stress, 74 to <78 = severe heat stress and 78 and more = very severe heat stress. When the temperature is measured in °C, the formula changes to: THI = [0.8 × ambient temperature] + [(% relative humidity ÷ 100) × (ambient temperature − 14.4)] + 46.4}. Mader [32] stated that the Livestock Weather Safety Index (LWSI) classified heat stress in cattle based on a °C formula as follows: normal, ≤74; alert, 74 < THI < 79; danger, 79 ≤ THI < 84; and emergency, THI ≥ 84. Based on Mader et al.’s [32] classification and formula, [33,34] it was concluded that the optimum THI for cattle and buffaloes under Egyptian environmental conditions is 71–74. However, Marai et al. [35] modified the formula (in °C) for sheep and goats to: THI = db °C − {(0.31 − 0.31 RH) (db °C − 14.4)} where db °C is the dry bulb temperature (°C) and RH is the relative humidity (RH%)/100. Using this formula, the optimum THI for sheep and goats under Egyptian conditions was concluded to be <22.2 = absence of heat stress; with 22.2 to <23.3 = moderate heat stress: 23.3 to <25.6 = severe heat stress and above 25.6 indicating extremely severe heat stress.

THI does not include some variables affecting the severity of heat stress, in particular air movement and solar radiation. A temperature-humidity-velocity index (THVI) has been created that includes wind speed [36]. The THVI, therefore, improves on the THI in determining the heat stress severity, with the formula: THVI = (0.85 × DBT + 0.15 × WBT) × V − 0.058, DBT: dry bulb temperature in °C; WBT: wet bulb temperature in °C; V: air velocity, with levels as follows: ≤70 normal, 70–75 alert, 76–81 danger, and ≥82 emergency.

## 3. Data Collection and Meta-Analysis

The Time and Date weather site [37] was used to collect average maximum temperatures for each month over thirty years, 1985–2015, to examine which months had a maximum THI of >22.2 for sheep and >74 for cattle, as determined above [32,33,34,35]. Two Egyptian locations were investigated: Damanhur, Behira, (31.0425° N, 30.4728° E), a city close to the Mediterranean Sea, and Shibin Al Kawm, Menofia (30.5503° N, 31.0106° E), a city in the Nile delta with a hot desert climate. The aim was to determine whether the climatic conditions would be likely to cause sheep and cattle to experience heat stress. These two locations were chosen to represent the north and middle region of the Nile delta, respectively, which is where most livestock are kept. The results indicated that in 66.67% of months sheep were exposed to maximum temperatures indicating heat stress in Damanhour and Shibin Al kawm, while for cattle, the proportion was 25% in Shibin Al kawm only. Then the predicted increase in temperature as a result of climate change between 2011 and 2067 (on average) that was reported in a previous review was added [37]. This predicted that the proportion of months increased from 66.67% to 75% in Shibin Al kawm when sheep would be exposed to heat stress, while in cattle it was predicted to be increased from 25% to 41.67%.

**Figure 1:**
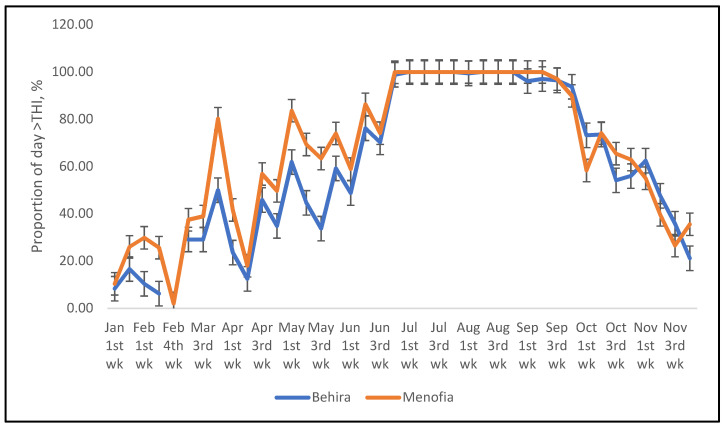
The proportion of half-hour periods in the day that sheep at the two locations (Behira and Menofia) in 2021 experienced THI greater than that believed to cause heat stress.

**Figure 2:**
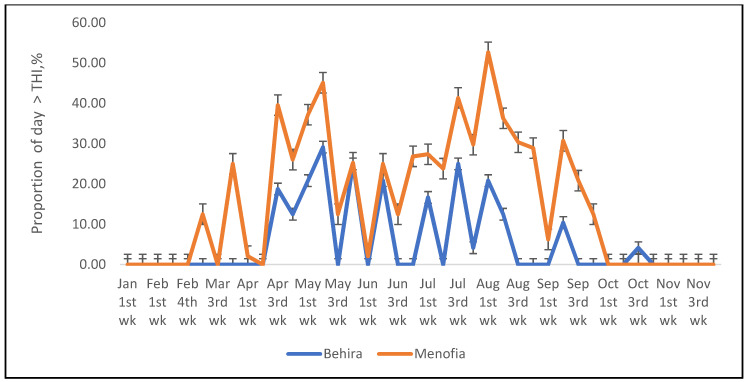
The proportion of half-hour periods in the day that cattle at the two locations (Behira and Menofia) in 2021 experienced THI greater than that believed to cause heat stress.

Because this dataset relied on monthly maximum values determined over 30 years, the Time and Date weather site [37] was also used to determine a more exact proportion of each day that sheep and cattle would experience a thermal environment which exceeded the critical THI’s in one year, 2021, also determined as above. Half hourly records of maximum temperature and humidity over 24 h of each day, were first used to determine the THI for each time period. Then the proportion of the total number of time periods for which the THI exceeded the critical value was determined and presented as a graph. The results show that sheep are exposed to heat stress-inducing thermal conditions for much of the summer period, 21 June to 21 September (Figure 1), however for cattle the period of exposure to temperatures exceeding the maximum THI was much less (Figure 2), but this difference between the species may be due to inaccuracies in the critical THI values adopted in this study.

**Figure 3:**
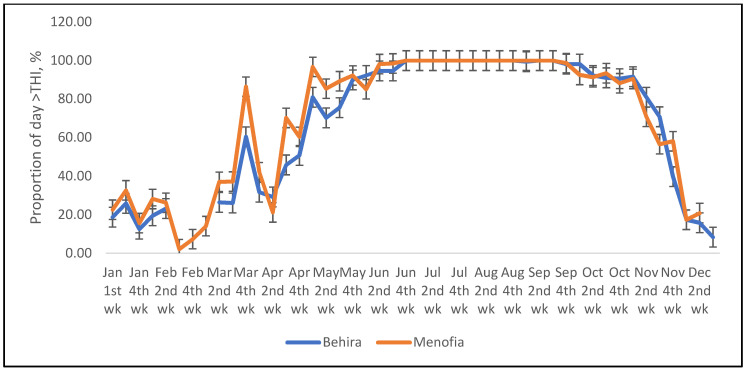
The predicted proportion of half-hour periods of the day that sheep at the two locations (Behira and Menofia) are likely to experience THI greater than that believed to cause heat stress, after addition of temperature increment expected from climate change [38].

**Figure 4:**
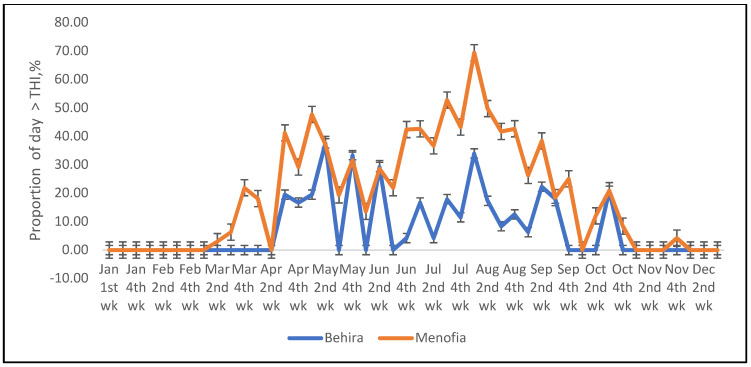
The predicted proportion of the half-hour periods of the day that cattle at the two locations (Behira and Menofia) are likely to experience THI greater than that believed to cause heat stress, after addition of temperature increment expected from climate change [38].

Similarly, the predicted increase in temperature between 2011 and 2067 from a previous review [38], was used to determine a predicted THI and proportion of time period per day sheep and cattle would experience heat stress at some point in each half-hour (Figure 3 and Figure 4). This maximum proportion of half-hour periods that sheep were expected to experience thermal stress then extended from May until October (Figure 3) and was similarly increased in cattle (Figure 4).

## 4. Animal Responses

Stress is “an environmental effect on an individual which overtaxes its control systems and results in adverse consequences, eventually reduced fitness” [39]. Considering this, we have adapted the heat stress definition proposed by Egyptian scientists [40]: the consequence of an animal activating its physiological mechanisms to maintain thermal balance, following or during exposure to high environmental temperature.

During heat stress, animals reduce their feed intake to reduce metabolic heat production, thus controlling the increase in core body temperature caused by digestion, which is an important protective mechanism. Affected animals also drink large amounts of water, at least five times the normal amount drunk under temperate conditions, which increases urination and mineral loss [41]. Estimating drinking water consumption by livestock is hard because it is influenced by temperature in the environment, humidity, the type of animal and its physiological status, dietary intake, the water content of the diet, and water quality. Models often only account for 35% of the variance [42]. Inadequate water supplies reduce feed intake and reduce productivity. An important initial model for the water intake of cattle is that of Winchester and Morris [43]. Since that time, slaughter weights of cattle have increased, and higher water intakes are likely. Sexson et al. [44] estimate that water consumption increased by 0.22–1.9 L/°C. However, it is not a linear increase but exponential, with the highest intakes at temperatures above 40 °C. A popular model for dairy cows is that of Appuhamy et al. [45], which explains 76% of the variation. It is based on dry matter intake, dry matter content, ambient temperature (linear and square functions), milk yield, and dietary Na and K.

Heat-stressed cows also exhibit standing estrous less commonly, and it is mainly at night when it becomes cooler when they are less likely to be observed by stock people [46]. Other responses to heat stress include increased body temperature, sweating, respiratory rate, panting >80 breaths per minute (double the 35–45 normal), and reduction in activity and fertility [30].

Birds respond to heat stress by extending their wings from their body, increasing respiration rate, potentially leading to exhaustion, in an attempt to reduce the heat load through water vapor cooling. Other strategies include looking for isolated places of lower temperature, moving away from each other, less activity, decreasing feed intake, and increasing water consumption [47].

When the body temperature of chickens increases due to an increase in the ambient temperature above 30 °C, biochemical and physiological changes occur which may harm essential body organs if prolonged [48]. These responses include increased respiratory rate, up from 25 times/min to 260 times/min, leading to decreasing level of carbon dioxide and an increase of bicarbonate in blood plasma [49], which lowers hydrogen ions concentration and increases plasma pH, in a disease called alkalosis.

## 5. Adaptive Mechanisms

Livestock adapts in order to survive and reproduce under difficult environmental conditions [50]. These occur through several adaptive mechanisms, either behavioural and/or physiological, that could adversely affect the production and performance of animals. Animals cope mostly with stress through behavioural responses [51]. The adaptive behavioural responses of livestock [51], are in feeding, defecation and urination frequencies, water intake, lying and standing time, shade-seeking behaviour, and drinking frequency. These responses differ according to animals’ experience, the duration and intensity of the stressor, animal physiological status, and environmental constraints [51]. Understanding the behaviour of livestock could help in improving the handling and welfare of farm animals affected by heat stress. Under extreme climatic conditions, animals often have to walk longer distances to find feed and water [52,53]. The motivation for this may be mitigated through the neuroendocrine system [54].

Acclimatization occurs with the aid of the endocrine system in two stages, acute and chronic, with the latter taking weeks for completion. During these phases’ changes in hormone secretion and alteration in the number of the receptors in the target tissues occur [55]. These neuroendocrine responses vary depending on the stressor, with chronic stressors stimulating endocrine responses which may not be sufficient to prevent morbidity and mortality, but more adaptive functions are evident during acute stressors [56,57].

The peripheral thermal receptors are stimulated under high temperature, which transfer suppressive nerve impulses to the hypothalamus that causes a decrease in feed intake [58], as the animals try to adapt by reducing the heat of digestion. Increased temperature also leads to a reduction in growth because of decreased anabolic action and increased catabolism [58], which could be due to the increased production of catecholamines and glucocorticoids.

The rise in body temperature activates the preoptic thermostatic area to increase heat loss in three different ways from the body, first via sweat glands by evaporation, secondly by stimulating the vasodilator nerves of the skin to increase the diffusion of blood to the body surfaces, and third through inhibiting the posterior hypothalamus sympathetic centers to unlock the vasoconstrictor tone in the skin’s blood vessels, therefore permitting more vasodilatation [59].

These thermoregulatory reactions increase respiration and sweating, leading to a disturbance in water metabolism through higher water intake and body water content with negative effects on protein, energy, and mineral metabolism. Physiological responses include enzymatic reactions, such as increased transaminase activities. Decreased hormonal secretion of insulin, T4, T3, and aldosterone is apparent, while cortisol increases [60]. These alterations in blood metabolites impair feed intake, feed efficiency, growth rate, milk yield, and reproduction [60].

In poultry, the loss of heat is not by sweating because of the lack of sweat glands. The process of temperature regulation is controlled in birds by the hypothalamus through two pathways; first: sensible heat loss that occurs through conduction, convection, fecal excretion, and egg production, which all function best in the thermal comfort zone. The comb and wattles size provides sufficient skin space through which heat is transferred from the body to the head. Second: insensible heat loss, with increased evaporative cooling from increased heart rate, blood flow to the skin, and peripheral blood vessels expansion. Evaporation occurs from the mucosal surfaces of the lungs, air sacs, and the area between the nasal openings and the tracheal base [47]. Air sacs in chickens help in heat exchange with the environment, especially during panting, which facilitates air circulation and increases heat loss through evaporation together with gas exchanges [61].

## 6. Heat Stress Effects on Livestock Maintenance Behaviours

### 6.1. Feed and Water Intake

Feed digestion is a heat source for ruminants, thus, under increased heat stress they exhibit reduced appetite, gut motility, and rumination [62,63], which reduces feed conversion efficiency in dairy cows [64]. This assists in decreasing heat production during hot climatic conditions [65]. In temperate climatic conditions, feed intake declines above 25–26 °C in lactating cows, with a rapid decline above 30 °C, and a 40% reduction at 40 °C [66]. This negative energy balance decreases body weight and body condition score [67], which may facilitate heat loss.

Water intake is dramatically increased during summer, for example, a two-fold increase in buffalo calves compared with winter (39.2 vs. 20.1 L/day) and a three-fold increase in relation to metabolic body weight (W^0.75^). This increases the vaporization of water through the skin and respiratory surface [68]. Omran et al. [33] reported higher consumption of water by cattle and buffalo in Lower Egypt than in Upper Egypt due to the higher THI there.

Goats are less susceptible to heat stress-induced reductions in their feed intake when the temperature rises above the UCT [69]. Chickens have been observed to reduce feed intake by 9.5% up to six weeks of age when temperatures increased from approximately 21 to 32 °C [70]. The reductions in feed intake led to decreased feed conversion efficiency and weight gain [71,72,73,74]. In laying hens, a 1 °C increase in temperature above the ideal temperature reduces feed consumption by 1.6%, and energy consumption by 2.3% [47]. At temperatures above 30 °C, feed consumption is reduced by 5%.

### 6.2. Defecation and Urination

The urination and defecation frequencies in livestock vary with many factors, including water and feed intake, type of feed, disease condition, immunity, environmental temperature, and stressors [51]. Exposure of goats to high temperatures decreases defecation and urination frequencies, but not the duration of urination [75]. Alam et al. [76] also indicated a significant reduction in the urination and defecation frequencies of black Bengal goats exposed to heat stress. This reduction in the urination and defecation frequencies in goats is associated with increased respiratory and cutaneous evaporation, causing severe dehydration [77]. Non-lactating Holstein cows exposed to heat stress also decreased urine output, but with an elevation in the urinary sodium excretion and a reduction in sodium in serum [78].

### 6.3. Lying and Standing Behaviours

Cows spend about half of their time lying down, but this is reduced by heat stress [79]. Standing maximizes heat loss through evaporation from the body surfaces and an escape from the hot ground surface [80]. When the heat load increases by 15% (THI = 60–70), standing time increases by 10% to enhance heat loss, by increasing the skin surface area exposed to air [81]. Therefore, the skin is surrounded by air to which most body heat is transferred by convection, reducing the conductive heat loss [82]. In addition, the animals transfer only a small part of their heat load through their feet to the ground because of the small area of contact [83]. Sprinklers from the floor or above the cattle, showers as cattle exit the milking parlour and overhead fans can be used on some farms to mitigate heat stress, which facilitates heat load reduction and increases lying down time [84].

In some situations, livestock has been observed to lie down for longer during heat stress: Shilja et al. [77] recorded that heat-stressed goats had longer lying times when simultaneously exposed to restricted feeding, probably because standing requires more energy than lying down. This indicates a requirement for adequate levels of nutrition to provide sufficient energy levels for animals to perform the behavioural responses that could help them adapt to heat stress [85]. However, if nutrition is adequate reports testify that ruminants spend most of their night lying ruminating, but in heat stress, this reduces to allow more standing [86,87]. The increased standing can lead to increased lameness, particularly when cattle stand for longer than 45% of the day. It has been estimated that the reduction in lying time could reduce milk production by 1.7 kg of milk yield per hour in resting time [88]. There is also increased stamping behaviour, particularly by the left limbs, which suggests the involvement of the right-brain-centered flight or fight reaction [89]. Cattle in heat stress have a particular stance, with a lowered head, backward-facing ears, and a vertical tail [89].

## 7. Heat Stress Effects on Livestock Sexual Communication and Reproduction

How temperature changes affect sexual communication in animals is not well understood. Sexual communication depends mainly on pheromone detection [90], which might be impaired by variation in environmental conditions [91,92]. High temperatures can enhance pheromone degradation, thus impairing communication between conspecifics (e.g., in ants: [93]). Sex pheromones persistence decreases during the higher evaporation rate at high temperatures [94]. Thus, the impairment of the chemically mediated sexual signals during high temperatures could have an impact on the reproductive success of animals. Not only is the release of the sexual signal affected, but also the olfactory receptors of the recipient are impaired by extreme temperatures [90].

There is also a positive correlation between the temperature dependence of the membrane and odour sensitivity in the chemosensory receptors [95]. Thus, temperature fluctuation not only changes the pheromone profile, so the receiver does not obtain the correct information about the mate, but it also affects the way the pheromone is perceived as the receptors are not able to detect the odour. Livestock species that normally rely on communication between conspecifics for reproduction, in particular cattle and sheep, may therefore experience reduced reproduction rates. In addition, cattle are less likely to display estrous behaviour during the hotter daytime hours, when stock people are around to see them, and time their artificial insemination. They are more likely to display it at night when stock people are not available to detect estrous. Further, during heat stress fewer estrous events where cattle will stand to be mounted are detected, which leads to a reduced conception rate.

In addition, heat stress can inhibit gonadotropin-releasing hormone and luteinizing hormone synthesis which are responsible for estrous signs and ovulation [96]. Any higher body temperature than 39 °C during pregnancy could negatively affect the developing embryo during the period from days 1–6 and lead to abortion. It can also lead to premature calving by 10–14 days, which reduces the viability of calves [97]. In males, heat stress can lead to a reduction in semen quality and quantity and testicular size [98]. In sheep, premature termination of pregnancy reduces the viability of lambs, especially since brown fat laid down in late pregnancy is diminished. In broilers, it has been reported that males are more sensitive than females to heat stress-induced infertility [99], whereas, in layers, heat stress led to delayed ovulation, in addition to a reduction in yolk quality and hatchability [100].

## 8. Heat Stress Effects on Production

Milk production can dramatically decrease in dairy cows, by up to 35%, during heat stress, through reduced feed intake [101]. High-producing dairy cows are likely to be more sensitive to heat stress as they emit more metabolic heat than low-producing ones [102]. Consequently, due to the increased metabolic heat production under heat stress, milk production declines [65]. Milk protein and fat contents declined in dairy cows when the temperature–humidity index reached 72 or above [103,104]. Similarly, in dairy goats [105] and buffaloes [106], milk composition was adversely affected by heat stress.

Meat-producing ruminants were also affected by heat stress, with reductions in body weight and meat quality [107,108,109]. Goats and sheep as small ruminants are more tolerant of hot and humid weather conditions than large ruminants [110]. Although sheep and goats can cope with different adverse environmental conditions, there is an upper level of tolerance, after which they lose adaptation to heat capabilities, in which mitigation is required. Beef cattle are also more sensitive than other meat-producing livestock as they are more exposed to radiant heat, with their large surface area, and they are fed on high-energy diets [111,112]. Therefore, body weight can decrease in beef cattle under heat stress due to the decrease in feed intake and feed conversion [48].

The exposure of chickens to heat stress leads to the use of energy to attain thermoneutral conditions instead of growth [113]. This reduces the weight gain and feed conversion ratio in broilers [114,115,116]. Laying hens are even more sensitive to heat stress and both egg quality and egg production decline [117], in part due to alterations in calcium metabolism and alkalosis [118]. When the temperature increases above the ideal temperature, egg weight decreases at a rate of 0.07–0.98 g/egg for every 1 °C rise. Above 30 °C, egg production may be reduced by 1.5% [47].

## 9. Strategies to Overcome the Effects of Heat Stress on Livestock

In 2015, the Paris Climate Change Convention recommended to “hold the increase in the global average temperature to well below 2 °C above pre-industrial levels…. recognizing that this would significantly reduce the risks and impacts of climate change”. Later in 2016, 94 out of 197 nations agreed to apply the Paris Agreement, and developed countries mobilized USD 100 billion for climate projects.

Three strategies have been identified that could help in reducing the impact of climate change on ruminant production: (a) new nutrition methods that could help in increasing feed efficiency, including identification and characterization of new feed resources and utilization of agricultural residues [119], (b) new technologies of animal breeding including gene editing that could help in the use of genetic resources, also selection from the full spectrum of international breeds by new breeding methods, and with better use of local breeds [119], and (c) maintenance and enhancement of animal biodiversity [120].

Nutrition management methods to mitigate heat stress include the addition of novel feed ingredients, such as betaine to improve diet composition, and the alteration in time or frequency of feeding and watering [121]. These can help in increasing energy and electrolyte intake, with better maintenance of water balance. These modifications have been studied in cattle [122,123], and poultry [124,125,126] and showed benefits. In dairy cows, appropriate nutrition through supplying them with high-energy feeds and bypass protein could help animals to maintain their productivity [127]. Providing additional essential micronutrients, including mineral mixtures and antioxidants could also preserve milk production better when the animal is sweating profusely [128]. Yeast supplementation may also reduce the effects of heat stress on dairy cow production [127]. Das et al. [129] deduced that feeding heat-stressed dairy cows with a diet containing 14% acid detergent fiber increases milk yield and fat content. Genetic variation in livestock responses to heat stress has been detected in several studies [130,131]. These can help in selecting breeds that have less sensitivity to heat stress [132], or breeds that have better adaptation either physically or physiologically to heat stress.

Mitigation measures can be classified into interventions related to farm management and structure. Management practices include livestock genotype diversification, using genotypes that are resistant to heat stress and can cope with climate change and improve the livestock farms’ sustainability [133,134]. Livestock integration with forestry also provides shade and reduces solar radiation and ambient temperature, with synergistic positive effects on soil and nutrient cycles, reducing soil degradation and chemical use [135,136]. However, this will take time to establish and is unlikely to be developed in time to cope with rising temperatures unless there is major government support. The periurban nature of much of Egyptian livestock production further complicates the development of agroforestry systems.

Management interventions could also help in improving fertility [128], by hormonal treatment, for example. Preventive vaccination for emerging animal diseases could also help in coping with climate change. In addition to these measures, developing meteorological warning systems could help in determining the most suitable mitigation method to reduce the impact of severe weather events and prevent livestock losses [137].

The main structural interventions for intensive and semi-extensive systems are improving building orientation, insulation, reflectance, shading and ventilation, with or without the use of water cooling [138]. Therefore, providing shelter adjusts the micro-environment to reduce the heat stress effects on milk production [139]. Cooling dairy cows subjected to heat stress allows heat exchange between a cow and its environment, which reduces a cow’s core body temperature [140]. Cooling by providing shade can decrease ventilation, whereas cooling by fans alone, or in combination with sprinklers, is better in minimizing heat stress’ detrimental effects on milk production, reproduction, and the immune system [127,128].

These adaptation and mitigation strategies are species-, and context-specific. However, there are some options that are expensive or involve intensive resource consumption [141]. Therefore, intensive research is required to identify the appropriate mitigation and adaptation strategies locally, especially in developing countries, in addition to policy implementation [17]. Identifying genes responsible for adaptation phenotypes can also help in better adaptation which can be reached by genetic characterization of livestock species [3].

## 10. Conclusions

The effects of heat stress are becoming critical for livestock production systems, especially during summer in Egypt. The present study showed that Egyptian livestock at two centers of livestock production in the country would experience heat stress in summer for extended periods of each day. Livestock responds to these changes by using different mechanisms to survive, but production efficiency is severely compromised by heat stress. Injury and death may even ensue if mitigation measures are not taken quickly. Alternative solutions are needed to achieve food security in Egypt, which are likely to include reduced reliance on ruminant livestock for meat and milk production.

## Figures and Tables

**Table 1:** The lower and upper critical temperatures for different animal species.

Animal Species	Critical Temperature, °C		References
	Lower	Upper	
Dairy cows	−12/−1 ^a^	24	[19]
New-born dairy calf	8–10	35	[19]
One day old chicken	32	35	[19]
Finishing broiler	16	26	[19]
One day old turkey	35	38	[19]
Finishing turkey	16	26	[19]
Laying hens	16	27–29	[19]
Sheep	12	25–31	[24]
Goat	9 [20]	25–30 [22]	[20,22]
Beef cattle	−7–15 [21]	21–27 [23]	[21,23]

^a^ −12 °C for Holstein and Brown Swiss cows, −1 °C for Jersey cows.

## Data Availability

Not applicable.
